# Reproducibility of Published Randomized Controlled Trial Results in Cardiovascular Surgery: Protocol for a Meta-Epidemiological Umbrella Review

**DOI:** 10.2196/93997

**Published:** 2026-10-06

**Authors:** Allen Li, Arshia P Javidan, Daniel J Niven, Henry T Stelfox, Dean A Fergusson, Mamas A Mamas, Prasad Jetty, Charles de Mestral, Risa Shorr, Subodh Verma, Munir Boodhwani, Derek J Roberts

**Affiliations:** 1 Division of Vascular Surgery and Division of Cardiac Surgery, Department of Surgery, University of Toronto, Toronto, ON, Canada; 2 Department of Critical Care Medicine, University of Calgary, Calgary, AB, Canada; 3 Department of Critical Care Medicine, University of Alberta, Edmonton, AB, Canada; 4 Division of Vascular and Endovascular Surgery and Cardiac Surgery, Department of Surgery, University of Ottawa, The Ottawa Hospital, Civic Campus, Room A-280, 1053 Carling Avenue, Ottawa, ON, K1Y 4E9, Canada, 613-798-5555, Extension 16268; 5 Keele University, Stoke on Trent, England, United Kingdom

**Keywords:** cardiovascular surgical procedures, randomized controlled trial, reproducibility, umbrella review, PRISMA, Preferred Reporting Items for Systematic Reviews and Meta-Analyses

## Abstract

**Background:**

Reproducible study findings are those that are shown to be identical or nearly identical when an original study is repeated using similar methods. Findings from many randomized controlled trials (RCTs) in cardiovascular surgery have not been reproduced or are small, statistically fragile, and potentially influenced by funding source. They also demonstrate variable end point selection and protocol adherence, all of which may influence reproducibility of their reported results.

**Objective:**

This study aims to assess the reproducibility of RCTs conducted in the most commonly studied disease states in cardiovascular surgery that compared at least one open surgical procedure to an alternative open, interventional catheter-based, or sham procedure or medical therapy.

**Methods:**

We will conduct a meta-epidemiological study of the reproducibility of RCTs using umbrella review methodology. We designed the study protocol using the PRISMA-P (Preferred Reporting Items for Systematic Review and Meta-Analysis Protocols) statement and published recommendations on conducting umbrella reviews. We will search MEDLINE, Embase, and the CENTRAL from their inception for systematic reviews published in the English language that included data from phase 3 RCTs or those with a clinically important primary outcome and compared an open cardiovascular surgery (performed in the most commonly studied disease states across these specialties) to either an alternative open, catheter-based, or sham procedure or medical therapy. Two investigators will independently review the individual RCTs from these systematic reviews and extract data and assess risk of bias (using the Cochrane Risk of Bias-2 tool), the fragility or reverse fragility indices (which provide a measurement of the statistical robustness of the trials), and the authors’ interpretation of the results (also referred to as spin). RCTs will be classified as the original study if it was the first chronological study (based on participant enrollment date) to evaluate the compared interventions and as reproduction attempts if they reevaluate the compared interventions in a different set of participants. The interpretation of a study’s results will be based on the findings of its primary end point and be used to assess reproducibility. We will also create multivariable regression models to determine whether the adjusted odds of reproducibility are independently predicted by 12 different variables.

**Results:**

We registered the study protocol but have not yet conducted the search. We aim to complete the study by the end of 2026 and submit for peer review by the summer of 2027.

**Conclusions:**

This study protocol may be used as a framework for future reproducibility research. The results will help guide which cardiovascular surgery practices warrant careful interpretation and which are in need of reproduction studies. They will also identify characteristics of an original RCT in this field that predicts future study reproducibility before reproducibility studies are published.

## Introduction

Reproducibility of clinical research is increasingly recognized as an essential part of the scientific method [1-3]. Although initial promising results of randomized controlled trials (RCTs) may lead to the adoption of interventions in clinical practice, these may not be reproduced in subsequent reproduction RCTs [4]. One common approach to evaluating reproducibility is to evaluate study results reproducibility [5]. These studies repeat an original study’s methods to see if an identical or close-to-identical result is obtained [5]. Recent studies examining “results reproducibility*”* in clinical research have estimated that more than half of reproduced studies report results inconsistent with the original study’s findings [3,6-9]. This may be due to statistical (ie, random chance or sampling variation) or clinical heterogeneity between published RCTs.

There are several other reasons for a lack of results reproducibility between original and reproduction RCTs. There is an incentive for authors to publish positive studies for career requirements or advancement and other reasons [10]. Even negative studies often manipulate language to support the effectiveness or safety of the treatment allocated in 1 arm of the study over the other [1]. This incentive may lead to the use of suboptimal study designs or statistical methods [2,11]. Critical analyses of reproducibility in clinical studies have also suggested that the lack of open data sharing after publication may be another driver of the lack of reproducibility [1].

Evaluating the results reproducibility of surgical RCTs is particularly difficult because of unique challenges influencing their design and conduct [2,12,13]. The performance of surgical procedures can differ between surgeons, surgical practice groups, and centers, making surgical RCTs difficult to standardize [14,15]. There is also a learning curve associated with the conduct of new and/or unfamiliar surgical procedures [15], which may introduce performance bias against novel or evolving procedures in RCTs [14,16]. Surgical procedures also frequently undergo progressive technical refinements; further, the devices and surgical implants that surgeons use are often continuously being improved by manufacturers and others, each of which makes it difficult to determine when new procedures should best be compared to existing interventions [15]. These technical and engineering advancements may influence the potential effectiveness of the procedure being compared in the control and comparison arms over time. However, it is unclear whether the above challenges truly influence the reproducibility of surgical RCTs, as a systematic assessment of reproducibility in surgical trials has not yet been performed.

Cardiovascular surgery is one area in which demonstrating reproducibility of study results is critical. Cardiovascular disease is the cause of ~32% of global deaths and ~15% or more of all health care expenditure in the United States and other high-income countries [17,18]. Cardiovascular surgery is the second most investigated area of clinical medicine based on the volume of RCT evidence, trailing only behind interventions provided in general internal medicine [19]. There have been numerous trials comparing treatment of cerebrovascular, coronary artery, cardiac valvular and electrical (eg, atrial fibrillation) heart disease, and peripheral artery disease, including both acute and chronic lower limb ischemia [20,21]. However, recent evidence has suggested that most RCTs in cardiovascular surgery are small, statistically fragile, and potentially influenced by their funding sources; they also demonstrate variable end point selection and adherence to study protocol, all of which may influence their results reproducibility [13,22].

One recent example of the importance of reproducibility in cardiovascular surgery research is the results obtained from 2 large meta-analyses of RCTs comparing drug-eluting and non–drug-eluting technology for the treatment of peripheral artery disease [23,24]. Although the original pairwise meta-analysis of aggregated trial data suggested that the use of drug-eluting technology increased the risk of all-cause mortality, a subsequent individual patient data meta-analysis failed to find an effect on mortality. However, until the latter meta-analysis was published, the US Food and Drug Administration issued a warning regarding its use, and many clinicians markedly decreased use of this intervention internationally, even though many RCTs suggested that use of drug-eluting technology improved mid-term target lesion revascularization and intervention patency in adults with different severities and anatomical locations of peripheral artery disease. Although the US Food and Drug Administration warning was subsequently removed, it did lead to a significant interval decline in the use of drug-eluting technology for peripheral artery disease and halted or slowed the conduct of some RCTs in this area [25-27].

We aim to conduct a meta-epidemiological study to assess the results reproducibility of RCTs studying the efficacy or effectiveness of cardiovascular surgery interventions using an umbrella review methodology. Umbrella reviews are useful to synthesize findings when a large number of systematic reviews and meta-analyses have been published on a specific topic or when the study question is more broad [28]. The study objectives are to (1) identify cardiovascular surgery interventions with and without reproducible RCT evidence and (2) identify characteristics of an original RCT in the field of cardiovascular surgery that predicts future study reproducibility before reproducibility studies may be published.

## Methods

### Protocol Development and Registration

This will be a meta-epidemiological study of the results reproducibility of RCTs in cardiovascular surgery (see Figure 1 for a schematic summarizing the study selection and review process). The study will be conducted using an umbrella review methodology to identify trials of interest given the large number of published RCTs in this clinical area. The methods we will use to conduct the umbrella review are based on previously published recommendations [29]. We developed this protocol using the PRISMA-P (Preferred Reporting Items for Systematic Review and Meta-Analysis for Systematic Review Protocols; Checklist 1) statement [30]. The protocol was registered with PROSPERO (International Prospective Register of Systematic Reviews) before study start (PROSPERO registration number: CRD420261303338).

**Figure 1.**
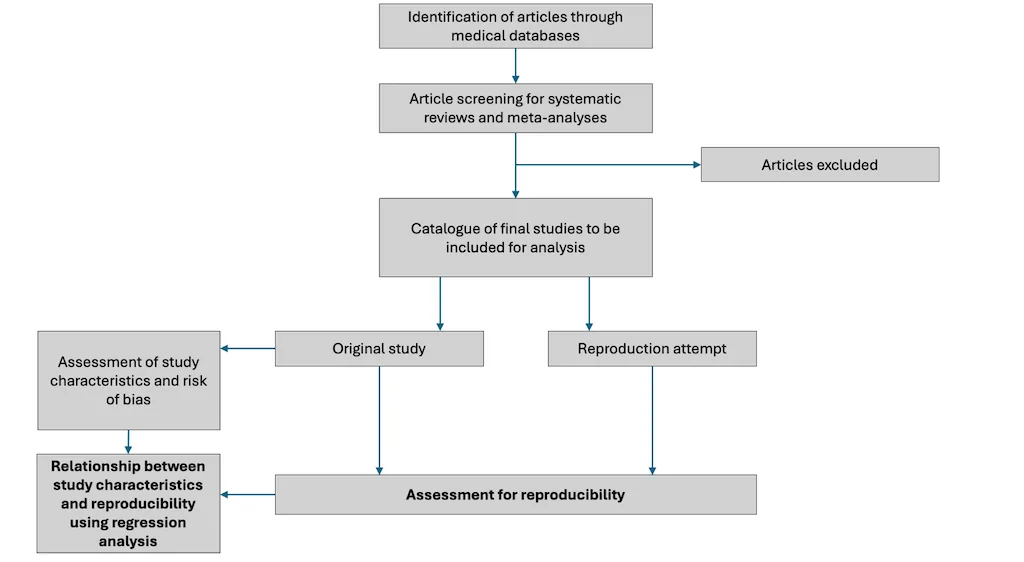
Schematic summarizing the study selection and review process during the umbrella review.

### Study Questions

The study questions are as follows:

1. In patients diagnosed with one of the most commonly studied disease states in cardiovascular surgery (see below), which open cardiovascular surgery interventions have evidence of results reproducibility overall and stratified by specialty (cardiac or vascular surgery) when compared to an alternative open, catheter-based, or sham procedure or medical therapy?
2. In RCTs comparing open cardiovascular surgery interventions to an alternative open, catheter-based, or sham procedure or medical therapy in the most commonly studied disease states in cardiovascular surgery, which characteristics of the RCTs predict results reproducibility overall and stratified by specialty?

### Eligibility Criteria

To identify RCTs, we will search for systematic reviews and then review their included RCTs to capture phase 3 RCTs (defined as a nonpilot trial that does not examine feasibility or surrogate outcomes and rather evaluates the effectiveness of a surgical treatment on clinically important outcomes) or those with a clinically important primary outcome (defined as a patient-important outcome that may affect morbidity, mortality, quality of life, or patient-reported outcomes, as determined by 2 independent investigators) that compared an open cardiovascular surgery performed in one of the most commonly studied disease states in cardiovascular surgery to an alternative open, catheter-based, or sham procedure or medical therapy. We will only include RCTs focusing on the most commonly studied disease states in cardiovascular surgery to allow for study feasibility given the expected large number of RCTs published in the cardiovascular surgery space. The definition of a procedure will be based on the American College of Surgery definition of surgery: *“*a procedure performed for the purpose of structurally alternating the human body.” We will only include RCTs published in the English language focusing on the most commonly studied disease states in cardiovascular surgery to allow for study feasibility given the expected large number of RCTs published in the field. The inclusion and exclusion criteria are summarized in detail in Textbox 1.

Textbox 1. **Inclusion and exclusion criteria.**

**Inclusion criteria**

- Systematic review or meta-analyses that included phase 3 randomized controlled trials (RCTs) or those with a clinically important primary outcome (as opposed to a surrogate or feasibility outcome) that enrolled adults (mean or median age 18 years or older) and compared at least 1 open cardiac or vascular surgery performed for one of the most commonly studied disease states in these surgical specialties in an operating room to another intervention (which can be an alternative open, interventional catheter-based, or sham procedure or medical therapy).

**Exclusion criteria**

- Studies that were not a systematic review or meta-analysis that included phase 3 RCTs or RCTs with a clinically important primary outcome.
- Studies that were scoping, realist, narrative, or other types of reviews.
- Systematic reviews where procedures were performed in nonoperating room settings (eg, outpatient angioplasty centers).
- Systematic reviews that were not published in the English language (for feasibility reasons).
- Systematic reviews published only as an abstract.
- Cochrane systematic reviews where a more updated Cochrane review had been done.

### Most Commonly Studied Disease States in Cardiovascular Surgery

To identify the most commonly studied disease states in cardiovascular surgery, we reviewed relevant literature on procedural rates in North America and the United Kingdom and consulted information sources released by the major governing societies in cardiac and vascular surgery (American Association for Thoracic Surgery, the Society of Thoracic Surgeons, the Society for Vascular Surgery, and the European Society for Vascular Surgery) [31-37]. We then invited a group of international expert cardiac and vascular surgeons, an academic interventional cardiologist, and a stroke neurologist to review and suggest revisions to this list. These surgeons or experts included prior members of the executive committees of national and international surgical societies and were identified first through suggestions by colleagues and then through snowball suggestions provided by the initial cohort. This process determined that the most commonly studied disease states in cardiac surgery are coronary artery disease, valvular heart disease, electrical cardiac disease (atrial fibrillation or flutter and other cardiac dysrhythmias), and aortic root pathology or dissections. In vascular surgery, they are carotid artery stenosis, abdominal aortic aneurysms, and peripheral artery disease, including both acute and chronic (ie, intermittent vasculogenic claudication and chronic limb-threatening ischemia) ischemia.

### Information Sources and Search Strategy

We developed the search strategy with the aid of cardiac (SV, MB) and vascular (DJR, PJ, CdM) surgeons, interventional cardiologists (MAM), RCT (DAF) and clinical study reproducibility (HTS, DJN) methodological experts [3], and a medical librarian or information specialist (RS). Using a combination of MeSH or Emtree terms and keywords, we created a search filter covering the domains coronary artery disease, valvular heart disease, aortic root pathology and dissections, electrical cardiac disease, carotid artery stenosis, abdominal aortic aneurysms, and peripheral artery disease, including both acute and chronic limb ischemia [22,31,38-43]. This domain was then combined with 3 published and validated search filters designed to identify systematic reviews of RCTs in MEDLINE, Embase, and CENTRAL [44]. The search was then peer-reviewed by another medical librarian or information specialist as per the PRESS (Peer-Review of Electronic Search Strategies) guidelines [45] (see Table 1 for the final search strategy).

**Table 1.** Electronic bibliographic database search strategies.

Search theme	Search terms			
	Ovid MEDLINE and the Cochrane Database of Systematic Reviews		Ovid Embase	
	Exploded MeSH terms	Title and subject keywords	Exploded Emtree terms	Title and subject keywords
Coronary artery disease, valvular heart disease, aortic root pathology/dissections, electrical cardiac disease, carotid artery stenosis, abdominal aortic aneurysms, and peripheral artery disease, including both acute and chronic limb ischemia	Coronary artery disease/ exp coronary artery bypass/ exp aortic aneurysm, abdominal/ exp peripheral arterial disease/su arterial occlusive disease/su endarterectomy, carotid/ exp arrhythmias, cardiac/ arterial occlusive disease/su or endarterectomy/ or ischemia/su or lower extremity/su	cabg.tw,kf. (coronary arter* adj2 bypass*).tw,kf. ((aort* valve* or mitral* valve*) adj3 (repair* or replacement*)).tw,kf. aort* dissect*.tw,kf. (TBAD or TAAD).tw,kf. (abdomin* aort* adj2 aneurysm*).tw,kf. (thoracoabdomin* aort* adj2 aneurysm*).tw,kf. ((lower extremity or leg or lower limb*) adj2 revasculari?ation*).tw,kf. ((chronic or critical) adj3 (limb* or leg* or extremit* or foot or feet or toe or toes) adj3 (isch?em* or obstruct* or occlus* or steno* or block* or obliter* or thrombo*)).tw,kf. ((aortofemoral or aortobifemoral or femoral-distal or femoral distal or femoral-popliteal or femoral popliteal or femoral-tibial or femoral tibial or infrageniculate or suprageniculate or infrainguinal or lower extremity or lower limb or peripheral vascular) adj3 (arterial surg* or arterial bypass* or bypass* or bypass graft* or bypass surg* or graft* or intervention* or revascularization* or revascularization procedure* or vascular bypass* or vascular bypass surg* or vascular graft* or vein graft* or prosthetic graft*)).tw,kf. ((iliofemoral or femoral or femoral artery*) adj3 (endarterectom* or patch* or repair*)).tw,kf. carotid revasculari?ation*.tw,kf. (carotid adj2 (endarterectom* or stent* or stenos*)).tw,kf. (atrial fibrillation* or atrial flutter* or ventricular fibrillation* or ventricular flutter* or arrhythmia* or tachycardia or bradycardia).tw,kf.	coronary artery disease/ exp coronary artery bypass graft/ abdominal aortic aneurysm/ peripheral arterial disease/ endarterectomy/ limb ischemia/su carotid endarterectomy/ carotid stenosis/ heart arrhythmia/	cabg coronary arter* (with adj2 bypass*) aort* valve* (with mitral* valve*) (with adj3 repair* or replacement*) aort* dissect* TBAD TAAD abdomin* aort* (with adj2 aneurysm*) thoracoabdomin* aort* (with adj2 aneurysm*) lower extremity OR leg OR lower limb* (with adj2 revasculari?ation*) chronic OR critical (with adj3 limb* OR leg* OR extremit* OR foot OR feet OR toe OR toes) (with adj3 isch?em* OR obstruct* OR occlus* OR steno* OR block* OR obliter* OR thrombo*) aortofemoral OR aortobifemoral OR femoral-distal OR femoral distal OR femoral-popliteal OR femoral popliteal OR femoral-tibial OR femoral tibial OR infrageniculate OR suprageniculate OR infrainguinal OR lower extremity OR lower limb OR peripheral vascular (with adj3 arterial surg* OR arterial bypass* OR bypass* OR bypass graft* OR bypass surg* OR graft* OR intervention* OR revascularization* OR revascularization procedure* OR vascular bypass* OR vascular bypass surg* OR vascular graft* OR vein graft* OR prosthetic graft*) iliofemoral OR femoral OR femoral artery* (with adj3 endarterectom* OR patch* OR repair*) carotid revasculari?ation* carotid (with adj2 endarterectom* OR stent* OR stenos*) atrial fibrillation* OR atrial flutter* OR ventricular fibrillation* OR ventricular flutter* OR arrhythmia* OR tachycardia OR bradycardia
Systematic review filter	—^a^	(systematic review* or meta analy*).tw,kf. (systematic review* or meta analy*).pt.	—	(systematic review* or meta analy* or metaanaly*).tw. meta analysis/ or *“systematic review”/

a Not applicable.

### Data Management and Selection Process

Citations captured by the search will be uploaded into the Covidence systematic review software (Veritas Health Innovation), which will be used to remove duplicate studies. Subsequent screening will be done in 2 stages by 2 investigators independently and in duplicate. Two investigators will also screen the titles and abstracts of all identified citations independently and in duplicate. Those citations identified as potentially relevant by at least 1 investigator will then be reviewed in full by 2 independent investigators to determine final eligibility. Disagreements regarding study inclusion and exclusion will be resolved by consensus or the senior investigator (DJR) and if needed further discussion with other experts in cardiac (SV, MB) or vascular and endovascular surgery (PJ, CdM), interventional cardiology (MAM), and/or reproducibility research (DJN, HTS).

The included systematic reviews will be searched for included RCTs that reported sufficient data to be included in the study. Data will be collected from the included RCTs independently and in duplicate by 2 investigators using Microsoft Excel using a prespecified data extraction form piloted on a representative sample of 10 RCTs (Table 2 summarizes data to be collected). Disagreements between investigators regarding data extraction will be resolved by discussion or arbitration by the senior author (DJR). We will use standardized definitions of each study variable (ideally using validated and commonly used, reproducible definitions where available); conduct intermittent quality checks during data collection; and double-enter variables to ensure quality, transparency, reproducibility, and accuracy during data collection. To ensure data extractors understand and can apply each of the definitions, they will undergo training before beginning data extraction. They will also pilot the data extraction forms to ensure consistency and reliability before beginning data extraction.

**Table 2.** Data collection items.

Category	Data collection items
Study bibliographic information	Systematic review title and included RCT^a^ titles Country or countries and continent systematic review and included RCTs were conducted in Year of publication of the systematic review and included RCTs Clinical condition being investigated
Included RCT study design	Number of hospitals participating in the RCT Years of patient recruitment Patient baseline characteristics (age, comorbidities, smoking status, and surgical indications) Primary end point Study power Median or mean duration of follow-up (months) Source of study funding Impact factor of the journal publishing the RCT determined using the Clarivate Analytics Journal Citation Report Adherence to the CONSORT^b^ reporting guidelines
Risk of bias	Assessments performed using the Cochrane Risk of Bias-2 tool
Spin	Conclusion of study results in study abstract Conclusion of study results in the conclusion
Fragility or reverse fragility	Intention-to-treat sample size (control) Intention-to-treat sample size (comparison) Number of events of the primary end point (control) Number of events of the primary end point (comparison)
Reproducibility	Clinical context Primary end point (as defined by study authors or using our default study end points) Effect of intervention Original study or reproduction attempt Consistency with the original study, which may require meta-analysis when multiple reproduction attempts performed Benefit or risk effect size measurement and its surrounding 95% CI Methods reproducibility (whether the methods reported in the published results manuscript, the supplementary methods online, or the cited published protocol or clinical trials registry number would allow for the study to be repeated, as assessed by two independent investigators)

a RCT: randomized controlled trial.

b CONSORT: Consolidated Standards of Reporting Trials.

### Outcomes and Prioritization and Data Items

The primary outcome will be the prevalence of results reproducibility of published RCTs in cardiovascular surgery overall and stratified by specialty (cardiac or vascular surgery). The secondary outcome will be the adjusted association between original RCT characteristics and reproducibility. Study characteristics of interest will include the source of study funding (commercial or noncommercial), study sample size in the intention-to-treat analysis, study power, number of study sites (defined as number of hospitals participating in the study), whether the study was conducted in only 1 continent or multiple continents, “methods reproducibility” (ie, whether the methods reported in the published results manuscript, the supplementary methods online, or the cited published protocol or clinical trials registry would allow for the study to be repeated, as assessed by 2 independent investigators), the impact factor of the publishing journal corresponding to the year of publication of the study (determined using the Clarivate Analytics Journal Citation Report; Clarivate), the fragility or reverse fragility index (RFI; which provides a measurement of the statistical robustness of the trials, as is described below), the direction of the author interpretation of the results (also referred to as spin where the author or authors manipulate the language of their reporting, which does not align with the primary outcome), overall and individual component Cochrane Risk of Bias-2 (ROB-2) study risk of bias, the magnitude of the effect size between original trials and reproduction attempts, and adherence to the CONSORT (Consolidated Standards of Reporting Trials) reporting guidelines [46,47], and the differences in baseline patient characteristics across trials as a different baseline risk profile of the included patients may influence the reproducibility of trial results. Specifically, we will collect data on reported baseline age, comorbidities, smoking status, and surgical indications.

### Risk of Bias Assessment

The risk of bias of the included RCTs will be analyzed using the ROB-2 tool [48]. This tool analyzes the risk of bias arising from the randomization process, due to deviations from intended interventions or missing outcome data, and in measurement of the outcome and selection of the reported result [48]. Each trial identified will be assessed de novo by 2 independent investigators and then consolidated after disagreements are resolved (through consensus) to develop an overall summary of risk of bias across RCTs and component-based risk of bias rating for individual RCTs [49].

### Data Synthesis and Analysis of Reproducibility, Spin, and Fragility and Reverse Fragility Indices

Analysis of result reproducibility of articles will be performed using a modified methodology that has been reported previously [3]. Articles will be classified based on (1) the clinical context, (2) the primary end point used, (3) the effect of the intervention, (4) whether the study was an original study or a reproduction attempt, and (5) the consistency between the original study and reproduction attempts. The clinical context of a study will be classified by the interventions and disease process it is investigating (ie, endovascular compared to open surgery for treatment of an abdominal aortic aneurysm). In studies where the primary end point is not explicitly stated, the primary end points will be defaulted to major adverse cardiovascular events (defined as cardiac death, myocardial infarction, and stroke), all-cause mortality, and graft patency for cardiac surgery RCTs. The primary end point will be defaulted to either mortality and/or reintervention for abdominal aortic aneurysm trials, mortality and/or ipsilateral stroke for carotid artery intervention trials, and mortality and/or major adverse limb events or major (ie, above-ankle) amputation-free survival for peripheral artery disease (acute or chronic limb ischemia) RCTs. These end points were determined based on commonly reported outcomes across these RCTs and reporting guidelines from the Society for Vascular Surgery [50-52]. In settings where multiple primary end points are reported, each one will be recorded and used in separate assessments for reproducibility. Single and composite outcomes will be analyzed based on the definition of the original study. Definitions used for the primary end point will also be assessed as differences between primary end points may create a difference in a study’s results. If studies all differ in the prespecified end point, the default end points listed above will be used instead. As there is a possibility of an abundance of studies requiring the default end point to be applied, a sensitivity analysis of reproducibility will be done for studies where the primary end point was reported, and no default end points were used. The definitions used for the primary end point will also be assessed, as differences between primary end point definitions may create a difference in the study’s reported results. The time at which study outcomes were assessed will also be recorded and stratified, as cardiovascular interventions may demonstrate significant outcome differences in the postoperative period, but these benefits may be lost during long-term analyses. In these instances, follow-up studies using the same dataset of an index trial will not be considered reproduction attempts, but rather original studies (eg, a longer-term follow-up of an original trial). Studies will be considered reproduction attempts of these follow-up studies if they are also follow-up studies occurring around the same time point. All studies will be classified as an “original study” or a *“*reproduction attempt.” A study will be classified as an original study if it was the first chronological RCT to examine the efficacy and safety of the effects of the cardiovascular surgical intervention used for a specific indication in a certain anatomical location. Any subsequent studies that evaluated the same outcomes of the results of an original study by recomparing the same 2 interventions for a similar indication in a similar anatomical location will be considered a reproduction attempt.

Reproduction attempts will be classified as “consistent effect estimates,” “possibly consistent effect estimates,” and “inconsistent effect estimates*.*” For reproduction attempts to have “consistent effect estimates,*”* both the original and reproduction studies need to have the same overall effect interpretation (defined as the reproduction attempt study having the same direction of effect and containing the original trial’s point estimate within its 95% CI and excluding the null-value of 1). For reproduction attempts to have “possibly consistent effect estimates,” the reproduction attempt study must have the same direction of effect and contain the original trial’s point estimate within its 95% CI, but this interval may include the null-value of 1. Finally, for reproduction attempts to have “inconsistent effect estimates,” the reproduction attempt must exclude the point estimate of the original trial from its 95% CI and have an opposite direction of effect. The above comparisons may require meta-analysis when there are more than 1 reproduction attempt RCT. In these situations, data will be combined using fixed effect or DerSimonian and Laird random-effects models depending on the extent of clinical heterogeneity as determined by 2 authors independently and in duplicate [53].

It is important to take into consideration that the method of statistical analysis conducted by a study may affect its interpretation and generate a degree of heterogeneity in reproducibility. As such, we will conduct a post hoc sensitivity analysis of reproducibility in studies that used the same methods of analysis to see if the degree of reproducibility changed. We will also assess “methods reproducibility” of the original study publication (or its cited protocol contained within the supplemental methods, published within a journal, or in the provided clinical trials registry) by examining whether the methods reported in the published result manuscript, the supplementary methods online, or the cited published protocol or clinical trials registry would allow for the study to be repeated, as assessed by 2 independent investigators.

Spin refers to the use of specific reporting strategies or manipulation of language that can alter the interpretation of study results by a reader. This has been heavily analyzed across many domains of medical and surgical literature, including cardiovascular medicine and surgery. To date, however, these analyses have been limited solely to studies with statistically nonsignificant primary end points. We will use the methodology originally developed by Boutron et al [54] to identify spin and classify it using a modified tool by Nguyen et al [22] which has been previously successfully used by our group previously to assess the vascular surgery literature [22,54]. Briefly, spin will be assessed in the abstract and manuscript of each study and then classified using one of four previously reported criteria: (1) authors pivot on statistically significant secondary results in the form of focus on within-group comparisons, (2) authors interpret statistically nonsignificant or significant results of the primary outcomes to show treatment equivalence or safety, (3) authors emphasize the beneficial effect of the treatment with or without acknowledging the statistically nonsignificant primary outcome or emphasize the beneficial effect of an inferior treatment despite a statistically significant difference, and (4) authors used a strategy of reporting or language manipulation that does not fall into one of the aforementioned criteria. A study will be classified as exhibiting “spin” if one of these techniques is found in the study title, abstract conclusion, or body conclusion, as these are sections frequently used by readers to formulate brief interpretations of study results.

Fragility and reverse fragility are metrics that quantify the statistical robustness of clinical trials with dichotomous primary end points [39,42,55]. The fragility index (FI; for statistically significant trials) and RFI (for statistically nonsignificant trials) have previously been analyzed in cardiovascular surgery by our group and are calculated as the number of event conversions needed to change the interpretation of a study’s primary end point [40,42,56]. This is accomplished by creating two-by-two contingency tables from the intention-to-treat analysis and subtracting events from a study group and adding nonevents to the same group until a 2-tailed Fisher exact test changes from significant to nonsignificant (for the FI) or vice versa (for the RFI). This is then repeated for the other group with the lowest value between the 2 groups being the FI or RFI of the study. If a study had reported more than one primary outcome, the FI or RFI will be calculated for each end point. In studies where the loss to follow-up exceeds the FI or RFI, there lies the possibility that the results of the study may have changed if all patients were accounted for. This will be analyzed for each study to determine if a loss to follow-up greater than a study’s FI or RFI plays a role in its reproducibility alongside the overall relationship between FI or RFI and reproducibility. The FI or RFI is limited in application to studies with dichotomous end points and cannot be applied to studies with continuous or time-to-event end points due to the nature of its calculation. The tools used to assess for spin and fragility or reverse fragility are summarized in Multimedia Appendices 1 and 2, respectively. Spin, FI, and RFI will all be collected in duplicate by 2 independent reviewers with discrepancies being resolved by a third independent study author.

### Statistical Synthesis

We will summarize continuous data using means and SDs or medians and interquartile ranges as appropriate. Dichotomous variables will be reported as percentages with 95% CIs estimated from the exact binomial distribution. We will also generate an exploratory multivariable logistic regression model to estimate whether the adjusted odds of reproducibility are predicted by a number of variables. These were predefined a priori to potentially include all of (1) the source of study funding (commercial vs noncommercial), (2) study sample size in the intention-to-treat analysis, (3) the reported study power, (4) number of study sites, (5) whether the study was international or conducted in one country, (6) whether the reported or cited methods were assessed to be reproducible, (7) the impact factor of the publishing journal corresponding to the year of publication of the study, (8) the FI or RFI, (9) spin, (10) the overall risk of bias as per the ROB-2, (11) the magnitude of the effect size between original trials and reproduction attempts, and (12) whether the authors adhered to the CONSORT reporting guidelines. We anticipate that some of these variables may exhibit collinearity and that the model will need to be reduced or simplified. We will assess for multicollinearity and overfitting by considering these concerns up front during model building through judgment and assessment using correlations between variables and testing during model development using common methods (eg, variance inflation factors). We will avoid transformations or statistical analyses in modeling study sample size in the intention-to-treat analysis by categorizing sample size into categories (very small, small, medium, large, and extra large). Missing data will be handled using complete case analysis.

Statistical analyses will be performed using Stata version 18 (Stata Corp). We will consider 2-sided *P*<.05 as significant.

### Confidence in Cumulative Evidence

This study is a meta-epidemiological analysis of the reproducibility of clinical trials. Therefore, an assessment of the strength of the overall recommendations is not pertinent.

## Results

The study received funding in June 2023 from the Canadian Society for Vascular Surgery to develop a detailed protocol. It subsequently received funding from the Division of Vascular and Endovascular Surgery at the University of Ottawa to support the conduct of the study beginning in May 2026. The projected dates of the conduct of the search, data collection, and data analysis will be from May to September 2026, September to December 2026, and January to March 2027, respectively. We anticipate that the results will be submitted for presentation at scientific meetings and peer review in the summer of 2027.

## Discussion

### Anticipated Principal Findings

The reproducibility of RCT results is critical in medicine and surgery and particularly in cardiovascular surgery given the associated potential morbidity and mortality of these procedures and their associated health care costs. This study will aim to appraise a wide body of cardiovascular surgical trials to identify reproducible and nonreproducible cardiovascular surgery interventions. We hypothesize that this study will find that only a relatively small number of cardiovascular surgery RCTs have been reproduced and that few cardiovascular surgery interventions are supported by reproducible evidence. We also hypothesize that original studies with smaller sample sizes and a higher risk of study-level biases, greater statistical fragility, and nonreproducible methods will be less likely to yield reproducible results. These findings may be used as a guide for trialists and consumers of cardiovascular research on future trial design and interpretation.

### Comparison With Prior Work

To our knowledge, only one prior evidence synthesis has been published to date that assessed the reproducibility of RCT results in medicine or surgery. Niven et al [3] conducted a scoping review to examine reproducibility within a cohort of RCTs examining clinical critical care research published in top medical and critical care journals [3]. They first searched the *New England Journal of Medicine*, *The Lancet*, and *Journal of the American Medical Association*, up to 2016, for RCTs examining the efficacy, effectiveness, or safety of therapeutic clinical practices among adults admitted to intensive care units. They then conducted a secondary search for RCTs examining these practices published in other high-profile general medical or critical care specialty journals. Overall, they identified that of 158 practices examined across 275 included articles, a reproduction attempt was identified for only 66 (42%; 95% CI 33%‐50%). Further, more than half of the clinical practices with a reproduction attempt demonstrated effects inconsistent with the original study (56%; 95% CI 42%‐68%).

### Strengths and Limitations

This study has several strengths. It incorporates a robust methodology (duplicate screening, a priori protocol registration, and input from a multidisciplinary team that spans all realms of cardiovascular and reproducibility research). It also incorporates advice from major governing societies across cardiovascular surgery and modern discussions surrounding clinical trial methodology that make it relevant to readers of research methodology and cardiovascular surgery.

However, our study also has important potential limitations. First, reproducibility will be assessed by analyzing the primary end point selected by the authors or by reporting standards of major governing societies. It should be acknowledged that secondary end points also offer significant insight and may alter the overall opinion of a study. Given that studies are not designed or powered for secondary end points, which can also be quite heterogeneous between studies or post hoc analyses of additional nonprespecified end points, they will not be included in the analysis of reproducibility. Second, the results reported by some reproduction RCTs may be different from the original trial because the reproduction study recruited a more comorbid population or the study was conducted in a different geographical region with potentially different clinical practice patterns. This study will attempt to account for this by analyzing the differences in baseline patient characteristics and setting or settings and its association with study reproducibility. Third, although it is possible that some RCTs may not yet be included in published systematic reviews and therefore missed by our search, we felt it necessary to use an umbrella review methodology instead of searching for individual RCTs for study feasibility reasons. Fourth, classifying the earliest published trial as the original attempt introduces possible bias as the first trial published may not necessarily estimate the true benefit or harm, potentially reducing estimates of results reproducibility. Fifth, reproducibility is assessed using point estimates and CIs which may not accurately estimate the true estimate as some trials may be underpowered or imprecise. Sixth, the assessment of fragility and reverse fragility is isolated to studies that use dichotomous end points as the Fisher test using event calculations cannot be performed on continuous end points. Finally, we will restrict our inclusion criteria to English-language RCTs that examined clinical practices within the most commonly studied disease states in cardiovascular surgery using information and procedural datasets derived primarily from North American and European professional organizations. Consequently, the applicability of these results to non-English language-predominant settings or less common cardiovascular conditions may be limited.

### Ethics and Dissemination Plan

As information collected in this study is publicly available and does not contain patient-level identifying data, ethics approval is not necessary at our institution. In an attempt to perform integrated knowledge translation (ie, where the created knowledge is more likely to be relevant to and used by knowledge users) [57], we involved key stakeholders in national cardiac (SV, MB) and vascular (PJ, CdM, DJR) surgery and interventional cardiology (MAM) societies as well as clinical study reproducibility (HTS, DJN) methodological experts [3] at the initial study planning phase, including the construction of clinical questions. This should ensure that the results of the study are more likely to be relevant to and used by knowledge users. We will also perform several end-of-project knowledge translation interventions by presenting results of the study at local and national cardiovascular surgery and medicine meetings and publishing study findings in relevant peer-reviewed journals.

### Conclusions

This study protocol may be used as a framework for future reproducibility research. The results will help guide which cardiovascular surgery practices warrant careful interpretation and which are in need of reproduction studies or further confirmatory research. They will also identify characteristics of an original RCT in this field that predicts future study reproducibility before reproducibility studies are published.

## Supplementary material

10.2196/93997 Multimedia Appendix 1 Assessment tool for the presence of study “Spin.”

10.2196/93997 Multimedia Appendix 2 Calculation of the fragility and reverse fragility index using 2 × 2 contingency tables.

10.2196/93997 Checklist 1 PRISMA-P checklist.

## Abbreviations

CONSORT: Consolidated Standards of Reporting Trials
FI: fragility index
PRESS: Peer-Review of Electronic Search Strategies
PRISMA-P: Preferred Reporting Items for Systematic Review and Meta-Analysis Protocols
PROSPERO: International Prospective Register of Systematic Reviews
RCT: randomized controlled trial
RFI: reverse fragility index
ROB-2: Cochrane Risk of Bias-2
