# Supplementary material for: Reproducibility of Published Randomized Controlled Trial Results in Cardiovascular Surgery: Protocol for a Meta-Epidemiological Umbrella Review
Source: JMIR Res Protoc. 2026 Oct 6;15:e93997. doi: 10.2196/93997 (PMC13641308; doi:10.2196/93997)
Supplement: Supplementary file 2 [file resprot-v15-e93997-s001.docx]

| **Strategy** | **Location in Manuscript** | | |
| --- | --- | --- | --- |
|  | **Study Title** | **Abstract Conclusion** | **Manuscript Conclusion** |
| Authors pivoted on statistically significant secondary results in the form of focus on within-group |  |  |  |
| Authors interpreted statistically nonsignificant results of the primary outcomes to show treatment equivalence or to rule out an adverse event |  |  |  |
| Authors emphasized the beneficial effect of the treatment with or without acknowledging the statistically nonsignificant primary outcome |  |  |  |
| Other, undefined, multiple strategies |  |  |  |

**Multimedia Appendix 1: Assessment Tool for the Presence of Study “Spin.”**
