# Supplementary material for: Reproducibility of Published Randomized Controlled Trial Results in Cardiovascular Surgery: Protocol for a Meta-Epidemiological Umbrella Review
Source: JMIR Res Protoc. 2026 Oct 6;15:e93997. doi: 10.2196/93997 (PMC13641308; doi:10.2196/93997)
Supplement: Supplementary file 3 [file resprot-v15-e93997-s002.docx]

**Multimedia Appendix 2: Calculation of the Fragility and Reverse Fragility Index Using 2x2 Contingency Tables.**

| **Trial 2x2 contingency table using the primary endpoint of the intention-to-treat analysis** | | | **Calculation of the fragility index** | | |
| --- | --- | --- | --- | --- | --- |
|  | **Event** | **No event** |  | **Event** | **No event** |
| Treatment | A | B | Treatment | A + FI | B – FI |
| Control | C | D | Control | C | D |
| Fisher exact test ≥0.05 | | | Fisher exact test >0.05 | | |
| **Trial 2x2 contingency table using the primary endpoint of the intention-to-treat analysis** | | | **Calculation of the reverse fragility index** | | |
|  | **Event** | **No event** |  | **Event** | **No event** |
| Treatment | A | B | Treatment | A – RFI | B + RFI |
| Control | C | D | Control | C | D |
| Fisher exact test ≥0.05 | | | Fisher exact test >0.05 | | |

Where FI indicates fragility index and RFI reverse fragility index.
